# Interaction of methotrexate, an anticancer agent, with copper(II) ions: coordination pattern, DNA-cleaving properties and cytotoxic studies

**DOI:** 10.1007/s00044-014-1074-1

**Published:** 2014-07-04

**Authors:** Justyna Nagaj, Paulina Kołkowska, Aleksandra Bykowska, Urszula K. Komarnicka, Agnieszka Kyzioł, Małgorzata Jeżowska-Bojczuk

**Affiliations:** 1 Faculty of Chemistry, University of Wrocław, F. Joliot-Curie 14, 50-383 Wrocław, Poland; 2 Faculty of Chemistry, Jagiellonian University, R. Ingardena 3, 30-060 Kraków, Poland

**Keywords:** Methotrexate, Copper(II) complexes, Plasmid DNA damage, Cytotoxic studies

## Abstract

The acid–base properties and the Cu(II) binding processes of methotrexate (MTX) were characterized by selected spectroscopic techniques and potentiometric measurements. The pH titration data showed that MTX behaves as a triprotic ligand. The deprotonation constants were determined for α-COOH and γ-COOH groups and (N1)H^+^ from the pteridine ring. Taking all the obtained results into consideration, a coordination pattern was proposed. The DNA-cleaving activity and reactive oxygen species (ROS) generation were investigated for both MTX and the Cu(II)–MTX system. The complex displayed a promising nuclease activity toward plasmid DNA in the presence of hydrogen peroxide. Interestingly, the induction of ROS, such as hydroxyl radicals, superoxide anions or singlet oxygen, was excluded and a different mechanism of DNA degradation was proposed. As MTX is now commonly used in anticancer therapy i.e. against lung cancer, basic cell-based studies were carried out to establish if its Cu(II) complex exhibits higher cytotoxic properties than the ligand alone. Activities of both compounds were also tested against colon carcinoma. Moreover, the determined values of IC_50_ were confronted with the cytotoxic activity of cisplatin.

**Electronic supplementary material:**

The online version of this article (doi:10.1007/s00044-014-1074-1) contains supplementary material, which is available to authorized users.

## Introduction

Methotrexate (MTX, (2*S*)-2-[(4-{[(2,4-diaminopteridin-6-yl)methyl](methyl)amino}benzoyl)amino]pentanedioic acid) is a folic acid antagonist and it has a therapeutic effect on many types of cancer cells. It is currently widely used as a major chemotherapeutic agent for human malignancies, such as acute lymphoblastic leukemia, lymphoma, osteosarcoma, and also breast, lung, head, and neck cancers (Yoon *et al.,* 2010). In the body, MTX is taken up by cells and tissues and then immediately metabolized to polyglutamate derivatives. Polyglutamates block the synthesis of purines and pyrimidines by inhibiting dihydrofolate reductase and several other folate-dependent enzymes. This blocking results in the disruption of DNA biosynthesis and is the basis of MTX chemotherapeutic action (Chibber *et al*., 2012). Tumor cells require about tenfold higher concentration of thymidine triphospate than healthy cells, and therefore they are more sensitive to the effects of antifolates (Navarro-Peran *et al*., 2005).

**Fig. 1:**
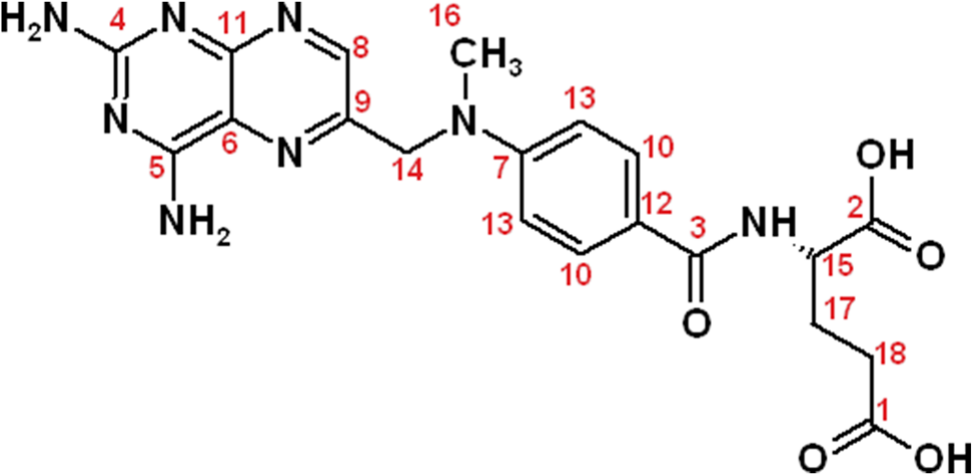
The molecular formula of MTX with atom numeration scheme used for ^13^C NMR spectra analysis

MTX is a methylated derivative of folic acid (Fig. 1). Its structure consists of a pteridine ring and dimethyl-p-aminobenzoic acid residue linked with glutamic acid. The coordination properties of this compound are not well characterized. Metal complexes of pteridines are rare since it is a highly π electron-deficient heterocyclic system (Kaim *et al*., 1999). On the other hand, the binding properties of glutamic acid, which forms thermodynamically stable complexes with a number of metal ions, are well characterized (Sajadi, 2010; Naik *et al*., 2012).

Copper is an important metal ion and an essential constituent of our biological enzyme systems. It is proven that both in inflammatory conditions and during neoplastic diseases copper plasma concentration rises from 15 μM/L in normal to 22–26 μM/L in cancerous cells (Zowczak *et al*., 2001). Hence, it is possible that chemotherapeutic drugs have an opportunity to interact with endogenous copper. Therefore, the aim of this work was to: (1) investigate the coordination properties of MTX toward Cu(II) ions, (2) verify the ability of this complex to generate ROS and DNA damage, and (3) establish the potential cytotoxic effect of the Cu(II)–MTX complex against two cancer cell lines.

## Experimental

### Materials

Methotrexate, CuCl_2_ × 6H_2_O, TSP-d_4_ (trimethylsilyl propionate), D_2_O, DNO_3_, NaOD, and pUC18 plasmid DNA were obtained from Sigma-Aldrich Co, Germany. NaOH, HCl, and ethylene glycol were purchased from Merck KGaA, Germany. Calibration buffers at pH values 4.01 and 9.21 was received from Mettler-Toledo GmbH, Germany.

### Potentiometric measurements

Potentiometric titrations of MTX and its complexes with Cu(II) in aqueous solution in the presence of 0.1 M KCl were performed at 298 K under argon atmosphere using pH-metric titrations (Metrohm, 905 Titrando). The CO_2_ free NaOH solution was used as a titrant. The samples were titrated in the pH region 2.0–10.5 using a total volume of 1.5 mL. Changes in pH were monitored with a combined glass–Ag/AgCl electrode (Metrohm, Biotrode) calibrated daily by HCl titrations (Irving *et al.,* 1967). Ligand concentration was 5 × 10^−4^ M, and metal to ligand molar ratios of 1:1 and 1:4 were used. These data were analyzed using the SUPERQUAD program (Gans 1983). Standard deviations (σ values) quoted were computed by SUPERQUAD and refer to random errors.

### Nuclear magnetic resonance (NMR)

^1^H NMR and ^13^C NMR measurements were performed on a Bruker AMX-500 instrument (^1^H: 500 MHz). TSP (trimethylsilyl propanoic acid) was used as an internal standard. Samples were prepared in 500 µl D_2_O (99.95 %) and the final concentration was 10 mM and 40 mM for proton and carbon spectra, respectively. NMR spectra were recorded for MTX and Cu(II)–MTX system at pD (pH measured by electrode uncorrected for the isotopic effect) value 7.5, which after appropriate correction (Krężel and Bal, 2004) is equal to 7.4. Measurements were made for solutions at five different Cu(II)–MTX molar ratios 1:500 ÷ 5:500. The pD of samples was adjusted by adding small volumes of concentrated DNO_3_ or NaOD.

### Infrared spectroscopy (IR)

The room temperature infrared powder spectra were recorded using Bruker IFS-66 FT spectrometer. The scanning range was 4,000–400 cm^−1^ and the resolution was 2 cm^−1^. Spectra of MTX alone and the Cu(II)–MTX complex were registered in a transmission mode as KBr pellets.

### DNA strand break analysis

The ability of Cu(II)–MTX complex to induce single- and/or double-strand breaks in the absence or presence of H_2_O_2_ was tested with the pUC18 plasmid on 1 % agarose gels containing ethidium bromide. The buffered samples (phosphate buffer, pH 7.4) contained combinations of DNA (25 μg/mL) and the components of investigated systems (metal ion and/or antibiotic, H_2_O_2_). Concentrations of each substance are given in figure captions. The metal to ligand molar ratio 1:1 was used according to the complexes stoichiometry based on the potentiometric calculations. After 1 h of incubation at 37 ***°***C in the dark, the reaction mixtures were mixed with 4 mL of loading buffer (bromophenol blue in 30 % glycerol) and loaded on 1 % agarose gels containing ethidium bromide (Sigma-Aldrich), in TBE buffer (90 mM Tris–borate, pH 8.0; 20 mM EDTA). Gel electrophoresis was done at a constant voltage of 4 V/cm for 60 min. As a control for double-strand breaks, reference plasmid samples were linearized with *Eco*RI endonuclease. The gels were photographed and processed with a Digital Imaging System (Syngen Biotech, Wroclaw, Poland).

### Reactive oxygen species (ROS) generation measurements

The ROS generation measurements were carried out with NDMA (*N*,*N*-dimethyl-4-nitrosoaniline) and NBT (nitrotetrazolium blue chloride), a scavenger molecules commonly used in studies of hydroxyl radicals and superoxide anion generation, respectively. The experiments were followed at 25 °C on a Cary 60 spectrophotometer. The solutions of NDMA and NBT at final concentrations 20 μM were added to the samples containing 50 μM Cu(II), MTX and Cu(II)–MTX, in the presence of 50 μM H_2_O_2_, at pH 7.4 (0.2 M phosphate buffer). The generation of singlet oxygen was tested by gel electrophoresis in conditions described above (“DNA strand break analysis” section) with an extra addition of NaN_3_ (singlet oxygen scavenger (Franco *et al.,* 2007)) at final concentration 40 mM.

### Cytotoxic assay

#### Cell lines and culture conditions

CT26 cell line (mouse colon carcinoma, morphology: fibroblast, ATCC: CRL–2638) and A549 cell line (human lung adenocarcinoma, morphology: epithelial, ATCC: CCL–185) were obtained from professor Luis G. Arnaut group (Chemistry Department, University of Coimbra, Portugal). Cells were cultured in flasks in Dulbecco’s Modified Eagle Medium (DMEM) without phenol red, with 10 % fetal bovine serum (FBS) and with 1 % streptomycin/penicillin at 37 °C and 5 % CO_2_ in a humidified atmosphere. Cells were passaged at preconfluent densities, using a solution containing 0.05 % trypsin and 0.5 mM EDTA. All the cell culture fluids were purchased from IMMUNIQ (Poland).

#### Cytotoxicity study

The cytotoxic activity in vitro was evaluated by the MTT assay. The assay was carried out according to the well-known protocol (Slater *et al.,* 1963). For the screening experiments, exponentially growing cells were harvested and plated in 96–well plates at a concentration of 1 × 10^4^ cells/well. After 24 h of incubation at 37 °C under humidified 5 % CO_2_ allowing cell attachment, the cells in the wells were treated with tested compounds at various concentrations in the range from 1 to 100 μM. The compounds were predissolved in phosphate buffer (pH 7.4) and diluted in the respective medium with 1 % FBS.

Two different protocols of cytotoxicity evaluation were performed. In the first approach cells were treated with 200 μL of tested samples: CuCl_2_, MTX, Cu(II)–MTX, and cisplatin for 4 h at 37 °C under conditions of 5 % CO_2_. Then, solutions were removed, cells were washed with PBS (phosphate buffered saline, IMMUNIQ, Poland) and fresh relevant medium was added. Cells were incubated for 24 h at standard conditions and then cytotoxicity was estimated once more. Whereas, in the second approach cells were incubated with various concentrations of tested samples diluted in DMEM containing 1 % FBS for 24 h in standard conditions. After that time surviving fraction was determined by MTT assay.

#### MTT assay

Briefly, a solution of 3–(4,5–dimethylthiazo1–2–y1)–2,5–diphenyltetrazolium bromide (MTT, Sigma) was prepared at 5 mg/mL in PBS and was diluted 1:10 in DMEM without FBS. 200 μL of this solution was added to each well. After 4 h of incubation at 37 °C in a humidified incubator with 5 % CO_2_, the medium/MTT mixtures were removed, and the formazan crystals formed by the mitochondrial dehydrogenase activity of vital cells were dissolved in 100 μL of DMSO:CH_3_OH dilution (1:1). The absorbance of soluble product was read with a microplate reader (Infinite 200 M PRO NanoQuant, Tecan, Switzerland) at 565 nm.

#### Data analysis

Cell viability was calculated using cells treated with DMEM containing 1 % FBS as control. Cell surviving fraction (%) was calculated using the formula: S/S_0_ (%) = [abs_565nm_ of treated cells/abs_565nm_ of untreated cells (control)] × 100. Each experiment was done in triplicate and was repeated at least twice. The inhibitory concentration (IC) values were calculated from a dose–response curve. IC_50_ values were determined from the fitting curve by calculating the concentration of agent that reduced the surviving fraction of treated cells by 50 %, compared to control cells. IC_50_ data are expressed as mean values ± standard deviation (SD) and they are the average of two independent experiments, done in triplicate.

#### Fluorescence microscopy

Viable and dead cells were detected by staining with AO (5 mg/L) and PI (5 mg/L) for 20 min and examined using fluorescence- inverted microscope (Olympus IX51, Japan) with an excitation filter of 470/20 nm. Photographs of the cells after treatment with the tested compounds were taken under magnification 20.00×.

## Results and discussion

### The acid–base chemistry of methotrexate

MTX molecule contains a 2,4-diaminopteridine ring and *N*,*N*-dimethyl-p-aminobenzoic acid residue linked with glutamic acid by a peptide bond (Fig. 1). It exists in water solution in a fully protonated form as a H_3_L ligand. The acid–base properties of the moieties, which can be deprotonated with a rise of pH value, were determined using potentiometric measurements (Table 1). The first two obtained p*K*_a_ values: 2.89 and 4.56 correspond to the deprotonation of carboxylic groups from glutamic acid, α-COOH and γ-COOH, respectively (Poe, 1973, 1977; Meloun *et al.,* 2010). The highest value of p*K*_a_ = 5.65 corresponds to the deprotonation process of the heterocyclic nitrogen (N1)H^+^ from the pteridine ring. The resulting p*K*_a_ values are quite consistent with the literature data. They have been first determined by Poe (1973) using potentiometric and spectrophotometric titrations, as 3.36, 4.70, and 5.71, respectively.

**Table 1:** Potentiometric parameters for MTX and its Cu(II) complexes

Ligand/complex	Logβ^a^	p*K*_a_^b^
H_3_L	13.10 (4)	2.89
H_2_L	10.21 (3)	4.56
HL	5.65 (3)	5.65
CuHL	8.82 (6)	–
CuL	4.01 (3)	4.81
CuH_-1_L	−2.32 (3)	6.33

^a^nH^+^+L^m−^ ↔ H_n_L, statistical errors on the last digits of stability constant are given in parentheses. Overall stability constant (β) expressed by equation β_HnL_ = [H_n_L^(m–n)−^]/[H^+^][L^m−^] describes a reaction

^b^Deprotonation constant (p*K*a) expressed by equation p*K*a = logβ (H_n_L^(m–n)−^) − logβ (H_n−1_ L^(m–n+1)−^)

### Investigation of the Cu(II)–methotrexate coordination mode

**Fig. 2:**
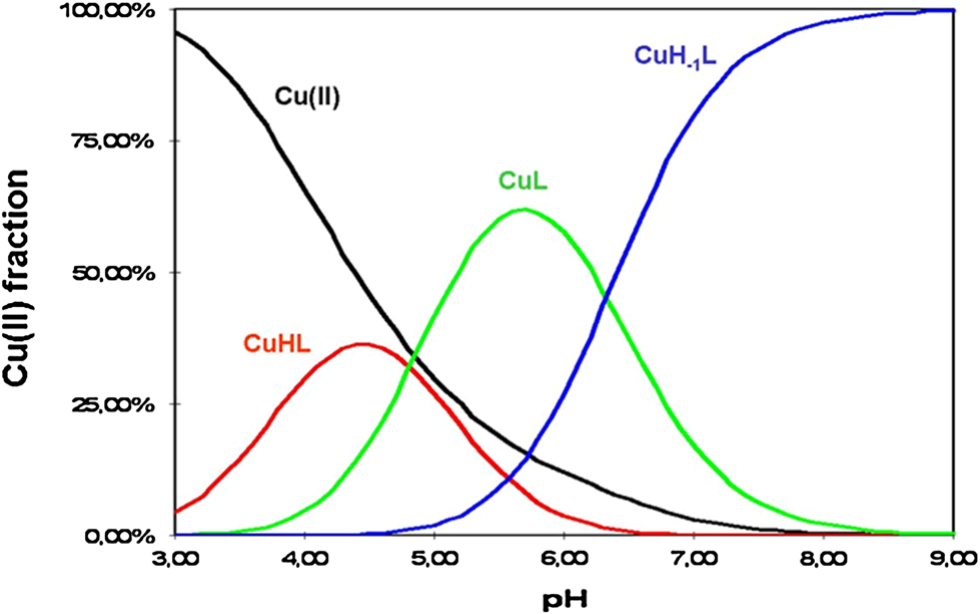
Species distribution diagram for the Cu(II)–MTX system

In order to obtain insight into the binding mode of MTX, the complex formation processes were studied by potentiometry, IR, and NMR spectroscopic techniques. These methods all together enabled verification of the type of donor atoms bound to Cu(II) ions and determination of the stability constants (Table 1). In the investigated pH range three monomeric complexes are formed: CuHL, CuL, and CuH_−1_L. Stability constants for *bis*-ligand complexes could not be established with certainty, therefore they were excluded from the accepted model. The binding process starts at pH 3.0 with the appearance of a CuHL form, as shown in the distribution diagram (Fig. 2). Considering the acid–base properties of the ligand, it is clear that in the presence of copper(II) ion the MTX molecule simultaneously loses two protons. The groups with the lowest p*K*_a_ values are the α-carboxyl and γ-carboxyl ones. It can be assumed that the Cu(II) ion binds to the oxygen atoms from both of them. With the rise of pH, the species distribution diagram reveals the occurrence of a new CuL form which reaches the maximum concentration at pH ~ 5.8. In that pH range deprotonation of (N1)H^+^ nitrogen takes place probably without its participation in the binding process. The last species, CuH_−1_L, is formed due to the forced dissociation of amide moiety caused by metal ion binding to this fragment of the studied molecule.

**Fig. 3:**
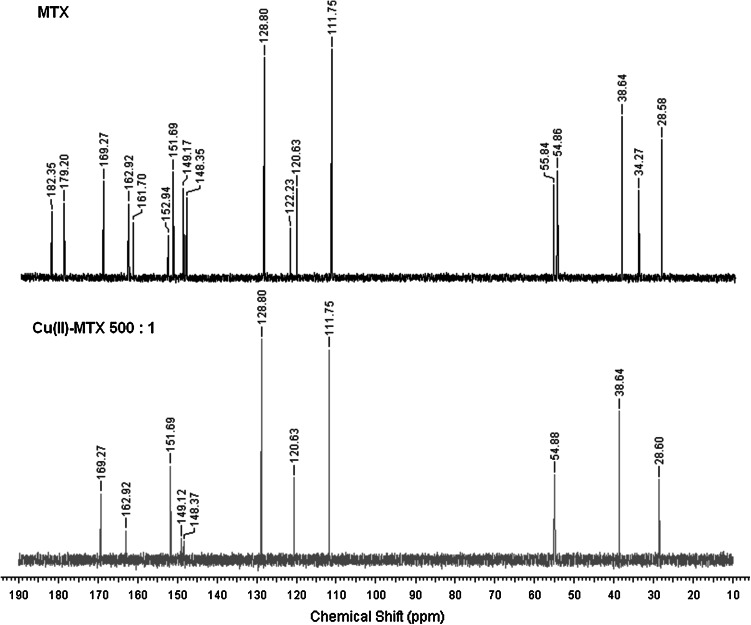
^13^CNMR spectra of MTX and Cu(II)–MTX solutions at pH 7.4

**Fig. 4:**
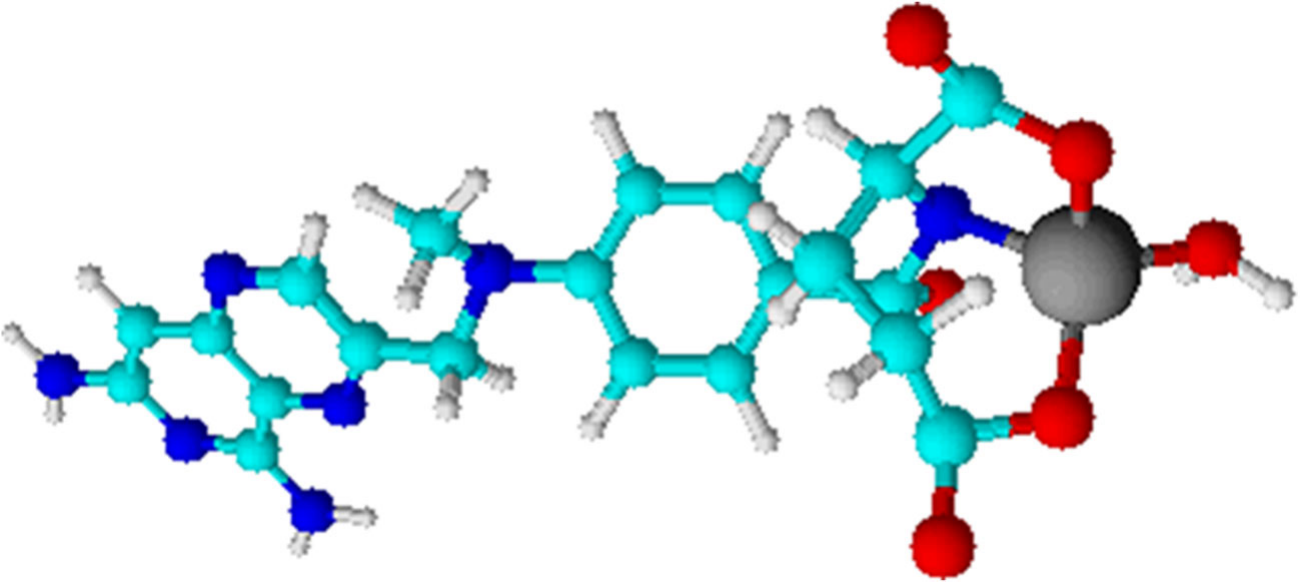
The model of Cu(II)–MTX complex existing at pH 7.5

These assumptions are supported by the NMR and IR results. Using NMR spectroscopy we could verify the type of donor atoms bound to the metal ion in solution. As in a number of other instances (Bertini and Pierattelli, 2004; Otting, 2010), also in this case the coordination of the paramagnetic cation causes a significant decrease of the intensity or even disappearance of the signals derived from the neighboring carbon atoms. Thus, the interaction of MTX with small amounts of Cu(II) solution (M:L 1:500) also results in vanishing of both carboxylic carbons and C_α_ signals from glutamyl residue (Fig. 3). The remaining peaks from glutamic carbon atoms and the neighboring C_C=O_ have a lower intensity. These findings support the model of coordination {α-COO^−^, γ-COO^−^, and N_amide_} deduced above (Fig. 4). The chemical shift values of MTX carbon atoms are collected in Table 2.

**Table 2:** The ^13^C NMR chemical shifts for MTX solution at pH 7.4

Carbon	δ [ppm]	Carbon	δ [ppm]
C^1^	182.3	C^10^	128.8
C^2^	179.2	C^11^	122.2
C^3^	169.3	C^12^	120.6
C^4^	162.9	C^13^	111.7
C^5^	161.7	C^14^	55.8
C^6^	152.9	C^15^	54.9
C^7^	151.7	C^16^	38.6
C^8^	149.2	C^17^	34.3
C^9^	148.3	C^18^	28.6

Assignments were made on the basis of Spectrum Database of Organic Compounds

**Fig. 5:**
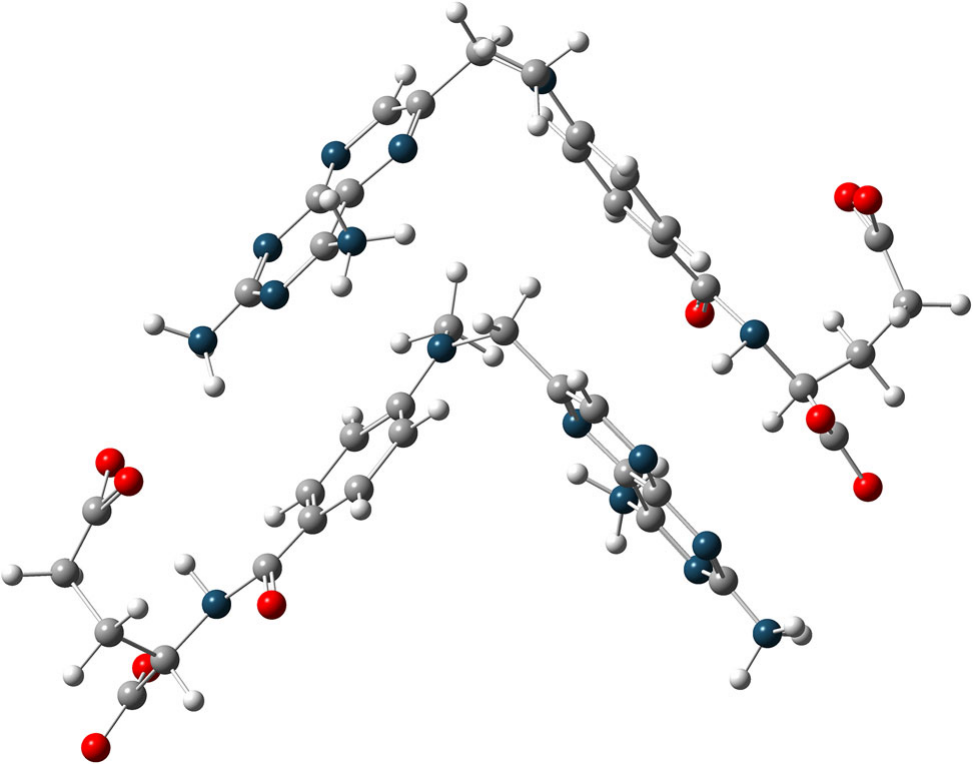
Proposed structure for MTX dimer on the basis of crystal data

Interestingly, the intensity of all ^13^C NMR signals from the pteridine ring also slightly decreases. The participation of this part of the molecule in the binding process does not fit the expected model. There could be one explanation for this phenomenon connected with the stacking interaction. The self-association of heterocyclic aromatic compounds has been observed for purines and pyrimidines, structurally related to MTX (Sigel and Griesser, 2005; Mitchell and Sigel, 1978; Dunger *et al.,* 1998). Therefore, this process can be expected in the studied case. MTX is known to aggregate, depending on the concentration and pH. However, the investigation of folates showed that these compounds do not form higher oligomers than dimers (Poe, 1973). According to this knowledge, at the neutral pH an MTX dimer consists of two molecules in a fully “stretched out” configuration. Consequently, both pteridine and *p*-aminobenzoate rings may participate in stacking interactions in a head-to-tail arrangement (Poe, 1973). This circumstance would be very helpful in the explanation of the disappearance of ^13^C NMR signals from pteridine moiety in the course of the present research. Chemical shifts are very sensitive to the environment. Looking at the proposed dimer structure, it is clearly seen that the pteridine ring is localized exactly above the *p*-aminobenzoate ring linked with glutamic acid (Fig. 5). Therefore, binding of copper(II) ions to carboxyl groups and amide nitrogen reduces the intensity of the signals of both the adjacent carbon atoms and pteridinic atoms.

The results obtained from FTIR experiments also support the proposed coordination mode. When comparing the solid state spectra of MTX and the Cu(II)–MTX system (Fig. S1), the most pronounced changes were recorded in the range of asymmetric stretching vibrations of COO^−^ groups (1700–1600 cm^−1^). These bands are not visible in the complex spectrum. Returning to the analysis of the ligand data, it is supposed that MTX exists in a zwitterionic form with a positive charge at two pteridine amino groups and a negative charge at carboxylate anions. An absorption band above 1700 cm^−1^ characteristic for the COOH group was not observed. However, there is a band in the range of 1690–1640 cm^−1^ which corresponds to the asymmetric stretching vibration of the COO^−^ moieties. Simultaneously, the band originating from the amino group vibrations does not appear. Instead, overlapped bands can be seen derived from the stretch vibration of carboxylate anions and asymmetric deformation of –NH_3_^+^. Such zwitterionic structure can facilitate the coordination of positive copper ion to the negative carboxylates.

### DNA damage and ROS generation by the Cu(II)–MTX system

**Fig. 6:**
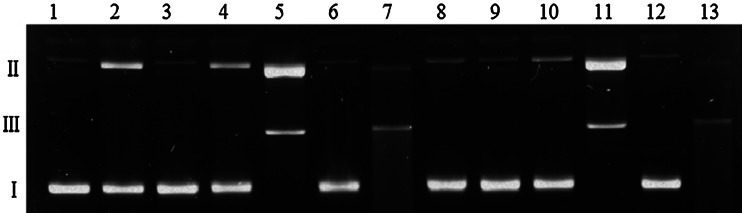
Agarose gel electrophoresis of pUC18 plasmid cleavage by MTX, CuCl_2_, and Cu(II)–MTX (1:1). *Lane 1*—untreated plasmid, *lane 2*—100 μM CuCl_2_, *lane 3*—100 μM MTX, *lane 4*—100 μM Cu(II)–MTX, *lane 5*—100 μM CuCl_2_ + 50 μM H_2_O_2_, *lane 6*—100 μM MTX + 50 μM H_2_O_2_, *lane 7*—100 μM Cu(II)–MTX + 50 μM H_2_O_2_, *lane 8*—50 μM CuCl_2_, *lane 9*—50 μM MTX, *lane 10*—50 μM Cu(II)–MTX, *lane 11*—50 μM Cu(II) + 50 μM H_2_O_2_, *lane* *12*—50 μM MTX + 50 μM H_2_O_2_, *lane 13*—50 μM Cu(II)–MTX + 50 μM H_2_O_2_

**Fig. 7:**
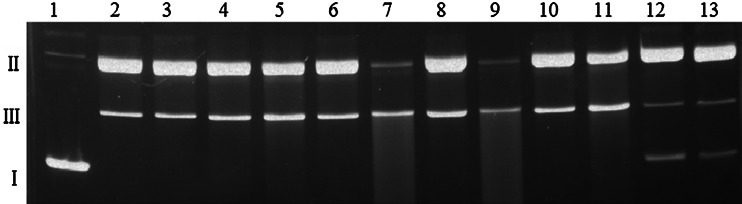
Agarose gel electrophoresis of pUC18 plasmid cleavage by Cu(II)–MTX (1:1) in the presence of 50 μM H_2_O_2_. *Lane 1*—untreated plasmid; *Even* *lanes*: + CuCl_2_ in concentrations: 1 mM, 500 μM, 100 μM, 50 μM, 25 μM, 5 μM; *Odd* *lanes*: + Cu(II)–MTX at the same, appropriate concentrations

In order to investigate the nuclease activity of the copper(II) complexes with MTX, pUC18 plasmid was used as the DNA substrate, and the resulting products were analyzed by an agarose-gel electrophoresis method. The cleavage activity was determined by measuring the conversion of supercoiled plasmid DNA (form I) to open-circular DNA (form II) or linear DNA (form III). The initial experiments show that the studied drug neither alone (Fig. 6, lanes 3, 9) nor in the presence of hydrogen peroxide (lanes 6, 12) is able to damage the DNA, regardless of the ligand concentration. Although Cu(II) ions alone (lanes 2, 8) and complexed (lanes 4, 10) yield some increase in the open-circular form II, significant changes in the plasmid structure are observed in the presence of H_2_O_2_ (lanes 5, 7, 11, 13). The obtained results demonstrate that complex-H_2_O_2_ (lanes 11 and 13) is the most efficient in plasmid degradation. As shown in Fig. 7, the Cu(II)–MTX-H_2_O_2_ system causes the cleavage of supercoiled DNA to its open-circular (II) and linear (III) form in a wide concentration range (from 5 μM to 1 mM). Moreover, these effects are accompanied by cutting the plasmid into shorter polynucleotide fragments, which is particularly evident on lanes 7 and 9. The quantity of the form II is in these cases negligible and streaks are the most visible. At a twice lower concentration of hydrogen peroxide, the plasmid destruction process is identical.

In order to gain some insight into the mechanism by which the complex-H_2_O_2_ system induces DNA cleavage, the ability to generate ROS was investigated. Most of the studied Cu(II) complexes have caused single- and double-strand DNA scissions by the oxidative mechanism in the presence of endogenous amounts of hydrogen peroxide (Suntharalingam *et al.,* 2012; de Hoog *et al.,* 2007; Devereux *et al.,* 2007; Szczepanik *et al.,* 2002; Jeżowska-Bojczuk *et al.,* 2002). It has often been evident that the presence of an antibiotic enhanced the oxidative activity of Cu(II) ions, and the resulting complex exhibited much higher potency toward ROS induction (Gaggelli *et al.,* 2010; Balenci *et al.,* 2009). The hydroxyl radicals detection is performed by monitoring the NDMA characteristic band at 440 nm on the electronic spectra. Generation of the **˙**OH radicals causes the decrease in the intensity of this band and can be measured in a time-dependent mode. The **˙**OH induction by the complex-H_2_O_2_ system was investigated in the conditions of gel electrophoresis experiments (50 μM concentration of both the complex and H_2_O_2_). However, only a slight decrease of the NDMA band was observed. The ability to generate superoxide anion by the complex-H_2_O_2_ system was also examined by performing a similar test with another reporter molecule-NBT. Likewise, the investigated system failed to induce this type of radicals. The next experiment was carried out using gel electrophoresis by adding sodium azide (singlet oxygen scavenger) to the reaction mixture. This procedure did not cause the inhibition of the cleavage reaction either.

Taken together, the obtained results suggest that the single- and double-stranded DNA cleavage mediated by complex-H_2_O_2_, does not occur by an oxidative mechanism. On the other hand, the same reactions performed without hydrogen peroxide do not result in plasmid degradation (Fig. 6, lanes 4, 10). This led us to propose that most probably the active species is copper-oxene or copper-coordinated hydroxyl radical (Sigman *et al.,* 1991; Baron *et al.,* 1936). The reactive species remain tightly bound to copper(II), thus preventing them from being deactivated by radical scavengers. A copper-oxene or a resonance hybrid of a copper(II)-hydroxyl radical species generates a deoxyribose-centered radical by C-1 hydrogen abstraction (Sigman *et al.,* 1991; Baron *et al.,* 1936), and is probably responsible for plasmid DNA cleavage in the studied case.

### In vitro cytotoxic studies

The anticancer activity of MTX, CuCl_2_, Cu(II)–MTX, and cisplatin against two selected cell lines: mouse colon carcinoma (CT26) and human lung adenocarcinoma (A549) were investigated. The evaluation of the cytotoxic activity of the compounds was carried out by the MTT assay, based on the ability of mitochondrial dehydrogenases in the viable cells to cleave the tetrazolium rings of MTT and to form dark blue membrane-impermeable crystals of formazan. The surviving fraction was determined by the relationship between the optical absorbance of dissolved formazan into a colored solution and the number of viable cell.

**Fig. 8:**
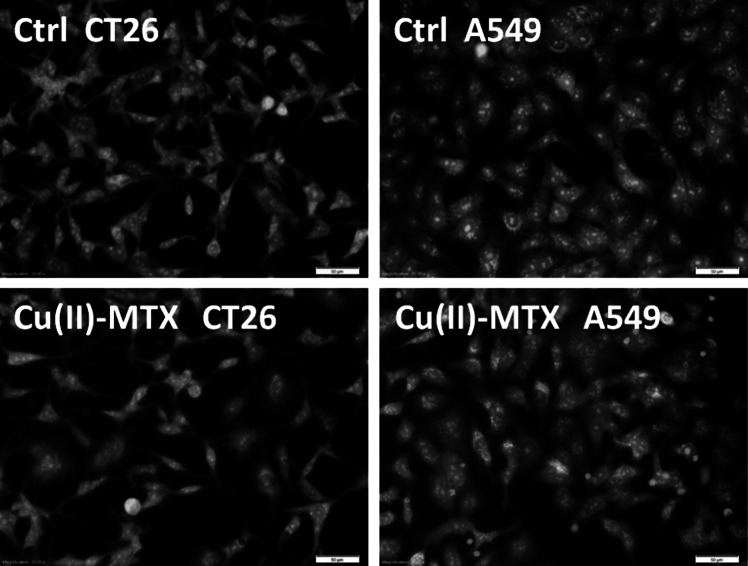
The selected photos (magnification ×20.00, *bar* 50 µm) of CT26 and A549cells after treated with the tested compounds (0.05 mM) for 24 h. The* green cells* with normal morphology are viable ones (AO+), while* round red cells* are dead (PI+)

The IC_50_ values were derived from dose–response curves and are summarized in Table 3. Cytotoxic study in vitro revealed that Cu(II)–MTX exhibits considerable toxicity toward both tested cell lines. The IC_50_ values obtained for the complex were in most cases lower than those for MTX and CuCl_2_. Generally, the greatest effect was observed on both cell lines after 4 h of incubation with the tested samples (Table 3). While after 24 h the impact of the complex on A549 line was similar (IC_50_: 188 μM), in the case of CT26 line the cytotoxic effect was dramatically lower (IC_50_: 1022 μM). These results indicate that A459 line is more sensitive for Cu(II)–MTX than CT26 cell line. It is noteworthy that all the tested compounds showed a significantly better anticancer activity than cisplatin (Table 3). Selected photographs of CT26 and A549 cell lines treated with the tested compounds are provided in Fig. 8. Cell viability was examined by counting the dead and alive cells stained with two fluorescent dyes. Accordingly, green cells with normal nuclei were treated as viable cells (AO+), while the red ones as dead (PI+). As can be noticed, Cu(II)–MTX caused a significant reduction only in the surviving fraction of A549 cell line (after 24 h of incubation time). This means that the investigated complex may exhibit selective biological activity toward only specific tumors. These studies indicate that Cu(II)–MTX exhibits biological activity toward specific cell lines and the cytotoxicity level is time dependent. The obtained results are preliminary and further investigations are needed to understand the molecular mechanism of cytotoxicity.

**Table 3:** IC_50_ values for MTX, CuCl_2_, Cu(II)–MTX, and cisplatin against CT26 and A549 cell lines after 4 and 24 h of incubation

	IC_50_ values [μM]^a^			
	4 h		24 h	
	CT26	A549	CT26	A549
MTX	258 ± 78	348 ± 32	460 ± 23	485 ± 12
CuCl_2_	360 ± 52	459 ± 32	423 ± 32	481 ± 11
Cu(II)–MTX	135 ± 17	151 ± 12	1022 ± 172	188 ± 52
Cisplatin	2200 ± 20	3150 ± 450	4990 ± 670	3850 ± 430

IC_50_ = concentration of drug required to inhibit growth of 50 % of the cancer cells (Strohfeldt *et al.*, 2008)

^a^Data are mean ± SD of three replicates each

## Conclusions

It was demonstrated that MTX interacts with Cu(II) ions and in aqueous solution it forms three monomeric complexes in a wide pH range. Moreover, basic biological in vitro studies were performed. In the presence of hydrogen peroxide the Cu(II)–MTX system displays nuclease activity, almost completely cleaving DNA. Most probably, the responsibility for the plasmid degradation processes may be attributed to the copper-oxene or copper-coordinated hydroxyl radical. Investigations of the anticancer activity showed that the complex generally displays higher cytotoxicity in vitro than the ligand and metal ion separately and is more selective against A459 cell line. As MTX is used in the treatment of lung cancer, our investigations demonstrated that complexation of MTX by Cu(II) ions results in its higher cytotoxicity. Moreover, in comparison to cisplatin, the Cu(II)–MTX system shows superior anti-tumor effects. MTX interacts with copper(II) ions forming complexes which display high DNA-cleaving propensity and promising cytotoxicity. The results presented herein can be helpful in the search for new cytostatic substances.

## Electronic supplementary material

Below is the link to the electronic supplementary material.

Supplementary material 1 (DOCX 120 kb)
