# Supplementary material for: Interaction of methotrexate, an anticancer agent, with copper(II) ions: coordination pattern, DNA-cleaving properties and cytotoxic studies
Source: Med Chem Res. 2014 Jul 4;24(1):115–23. doi: 10.1007/s00044-014-1074-1 (PMC4284383; doi:10.1007/s00044-014-1074-1)
Supplement: Supplementary file 1 — Supplementary material 1 (DOCX 120 kb) [file 44_2014_1074_MOESM1_ESM.docx]

**Fig. S1.** IR powder spectra of MTX (black) and Cu(II)-MTX (red).
